# *Cuatrecasanthus* (Vernonieae, Compositae): A revision of a north-central Andean genus

**DOI:** 10.3897/phytokeys.14.2520

**Published:** 2012-07-30

**Authors:** Harold Robinson, Vicki A. Funk

**Affiliations:** 1 Department of Botany, MRC 166, National Museum of Natural History, P.O. Box 37012, Smithsonian Institution, Washington, DC. 20013-7012

**Keywords:** Asteraceae, *Critoniopsis*, Ecuador, Neotropics, Peru

## Abstract

*Cuatrecasanthus* is native to Ecuador and Peru and although several unusual characters define the genus, such as single flowered heads and corolla throat (limb) divided to the base with lobes that are thickened at the margins, the members of the genus were not recognized as especially closely related until relatively recently. All six species are described, including two new to science (*Cuatrecasanthus kingii* H. Rob. & V.A. Funk, **sp. nov.** and *Cuatrecasanthus lanceolatus* H. Rob. & V.A. Funk, **sp. nov.**), and one new combination is recognized (*Cuatrecasanthus giannasii* (Stutts) H. Rob. & V.A. Funk, **comb. nov.**). A key is provided along with images of the types, SEM photographs of the leaf surfaces, a distribution map, and illustrations of the two new species. All species are given a preliminary conservation status of Data Deficient in regard to the IUCN Red List of Threatened Species.

## Introduction

The Andean genus *Cuatrecasanthus* H. Rob., native to Ecuador and Peru, is one of the most readily distinguished genera in the tribe Vernonieae. The combination of heads with one floret, corollas with the limb divided to the base into five scarcely distorted lobes, lobes with thickened margins, and ten-ribbed achenes is unique in the tribe. Another Andean genus, with similarly deeply cut corolla lobes, *Joseanthus* H. Rob., differs by its opposite leaves and many florets in each head. Although the distinctions of *Cuatrecasanthus* are clear, it has been subject to problems at the species level that have not been entirely resolved until the present effort to treat the genus for the Flora of Ecuador.

Given the distinctive characters of the genus, it is surprising that the first few species that were described were not recognized as relatives. The first member the group to be described was *Eremanthus jelskii* Hieron. from Peru. When *Vernonia flexipappa* was described by Gleason (1925), the relationship to *Eremanthus jelskii* was not recognized. Yet again, *Vernonia giannasii* Stutts (1980) was described without mention of the previously described relatives. It was Robinson and Kahn (1985), at the time of the description of *Vernonia sandemannii* (1985), who first recognized the relationship of the new species to the Hieronymus and Gleason species. At the time of the description of the genus *Cuatrecasanthus* (Robinson 1989) the three species were placed together, with *Vernonia giannasii* being treated as a synonym of *Cuatrecasanthus flexipappus*. The most recent studies have shown some errors in the 1989 treatment, with *Vernonia giannasii* proving to be a distinct species and two additional species needing description. The genus thought to contain three species now proves to contain six with all the additions being based on material from Ecuador.

Although material of *Vernonia flexipappa* was collected by Keeley in 1983, it was not reported in the DNA study of Keeley et al. (2007). Nevertheless, a position for *Cuatrecasanthus* in the subtribe Piptocarphinae near *Critoniopsis* Sch. Bip. is hypothesized on the basis of the woody habit, branching trichomes on the abaxial surface of leaves, and blunt-tipped sweeping hairs on the styles.

## Systematics

### Cuatrecasanthus

H. Rob., Revista Acad. Colomb. Ci. Exact. 17(65): 209 (1989).

http://species-id.net/wiki/Cuatrecasanthus

#### Type species:

*Vernonia sandemanii* H. Rob. & B. Kahn (=*Cuatrecasanthus sandemanii*(H. Rob. & B. Kahn)H. Rob.)

#### Description.

*Erect branching shrubs, scrambling shrubs or trees* (rarely vines) to 3.5 m tall; *stems* terete, striate, minutely pilose (pilosulous) with evanescent simple hairs or thinly tomentose; *pith* solid. *Leaves* alternate, petiolate; *blades* elliptical or lanceolate, base narrowly cuneate to attenuate, subchartaceous, margins entire to remotely subserrulate, narrowly recurved, apex usually sharply acuminate, adaxial surfaces pilosulous with simple non-septate, thick-walled trichomes, with numerous glandular dots, abaxial surfaces covered with thin whitish tomentum of prostrate myceliiform minutely branching trichomes; *secondary veins* 4–9 on each side of midvein, ascending basally at 45–60° angles. *Inflorescence* terminal on leafy stems, rounded corymbiform, branching alternate, with large foliaceous bracts only at lower primary nodes. *Heads* clustered and sessile in glomerules at ends of short branchlets (Figs 7C, 9B), individual heads cylindrical; *involucral bracts* ca. 15 in 5–6 gradate series (Figs 7D, 9C), inner bracts easily deciduous, outer bracts persistent; *receptacle* glabrous. *Florets* one per head; *corollas* lavender, outside minutely gland-dotted, distally sometimes pilosulous, basal tube narrow, ca. 2.5–4.0 mm long, throat lacking, lobes 5, linear, separated to base of limb, with somewhat thickened margins, not or scarcely distorted on drying (Figs 7E, 9D); *anther* thecae purple, with short papillose-fimbriate basal appendage, apical appendage ovate-oblong, ca. 0.5 mm long, glabrous; *style* base with stopper-shaped node, with thick-walled cells, sweeping hairs non-septate, obtuse to short-acute. *Achenes* prismatic, 10-costate (Figs 7G, 9F), surface sometimes fleshy, with numerous glandular dots, with few or no eglandular trichomes, with minute short-oblong raphids, base with broad annuliform carpopodium; *pappus* straw-colored, of 45–65 crowded rather persistent capillary bristles, about as long as corolla, barbellate, mostly some somewhat broadened and flattened distally, a few outer shorter bristles rather indistinct. *Pollen* ca. 40–45 µm in diam., spinulose, sublophate, tricolporate, with continuous perforated tectum between colpi.

**Figure 1.**
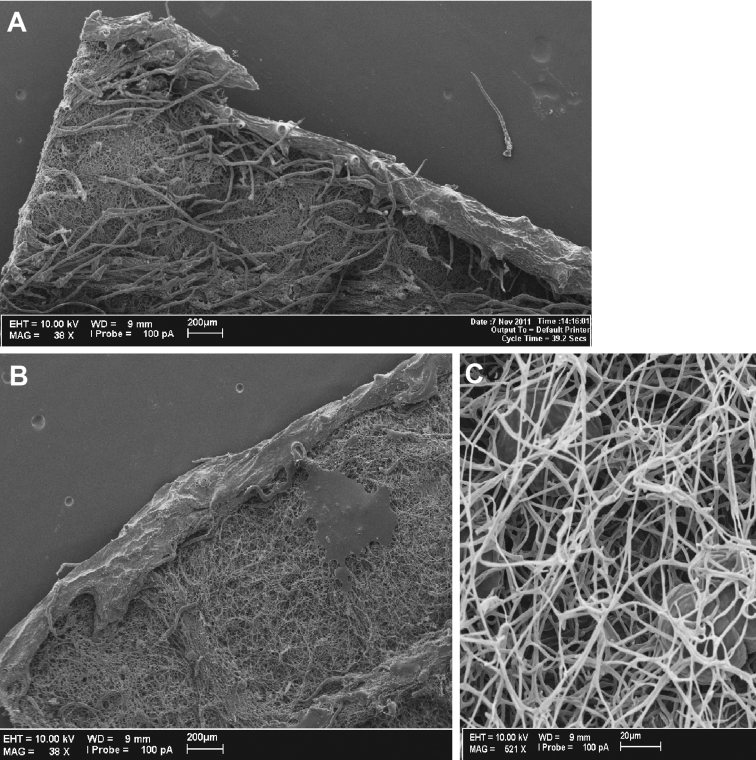
*Cuatrecasanthus* leaves: **A** *Cuatrecasanthus lanceolatus* showing projecting marginal tooth **B** *Cuatrecasanthus kingii* showing incurved tooth **C** Myceliform hairs on abaxial surface of leaf of *Cuatrecasanthus giannasii*.

**Figure 2.**
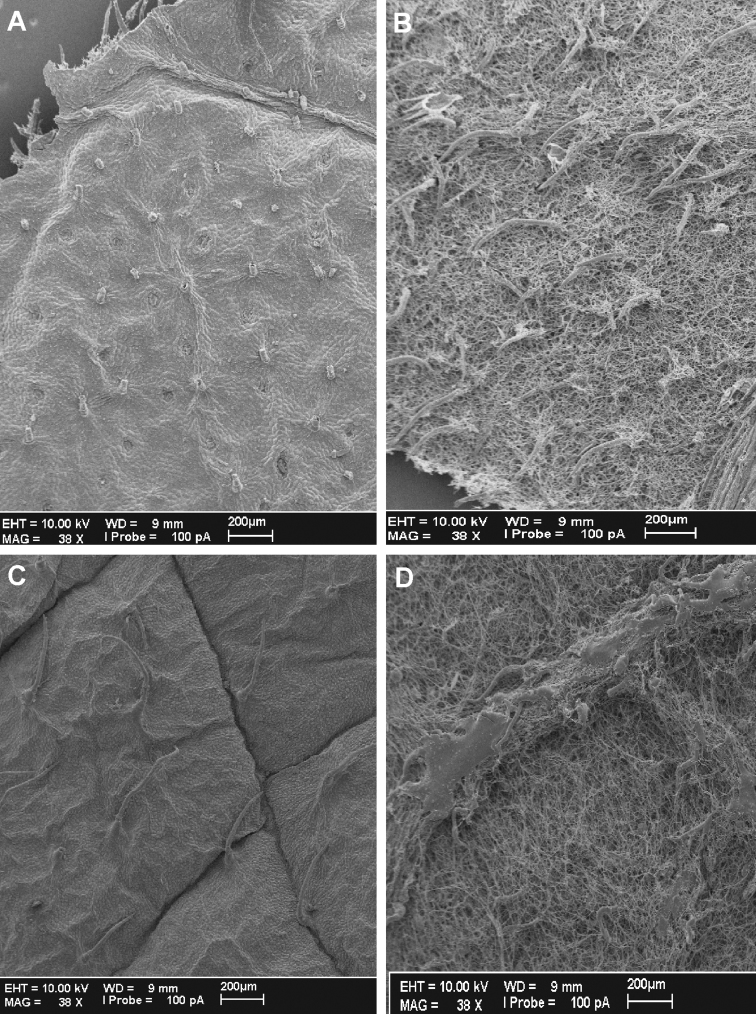
*Cuatrecasanthus* leaf surfaces: **A–B** *Cuatrecasanthus flexipappus*. **A** Adaxial surface **B** Abaxial surface **C–D** *Cuatrecasanthus giannasii* **C** Adaxial surface, showing deeply insculpate veins **D** Abaxial surface.

**Figure 3.**
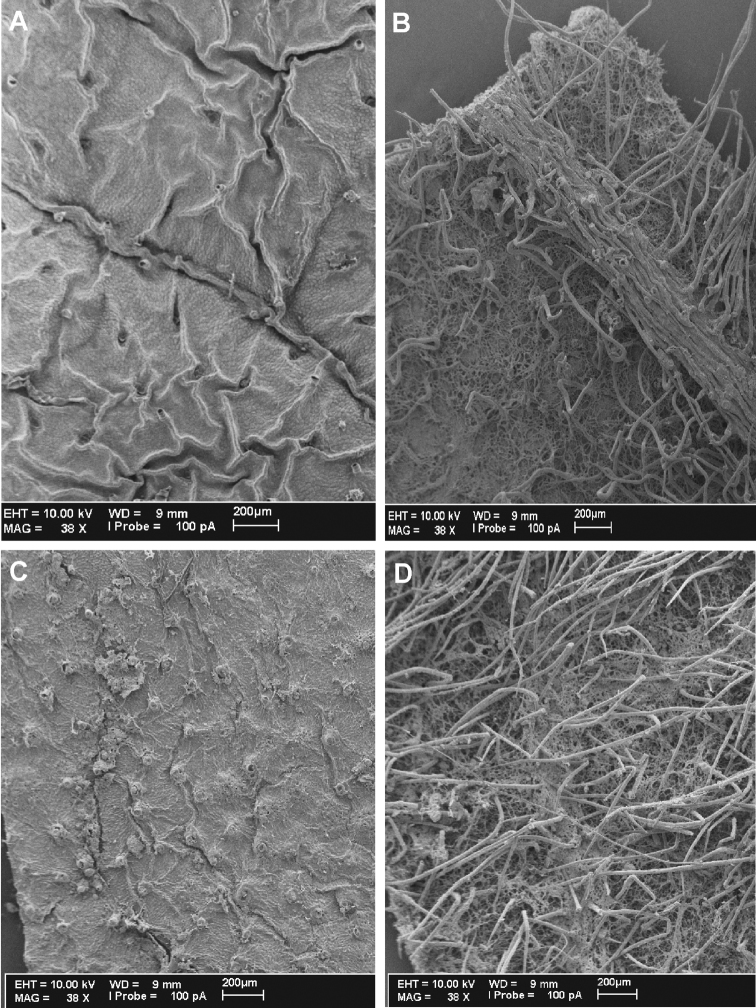
*Cuatrecasanthus* leaf surfaces: **A–B** *Cuatrecasanthus jelskii*. **A** Adaxial surface **B** Abaxial surface **C–D** *Cuatrecasanthus kingii*. showing veins even with surface **D** Abaxial surface.

**Figure 4.**
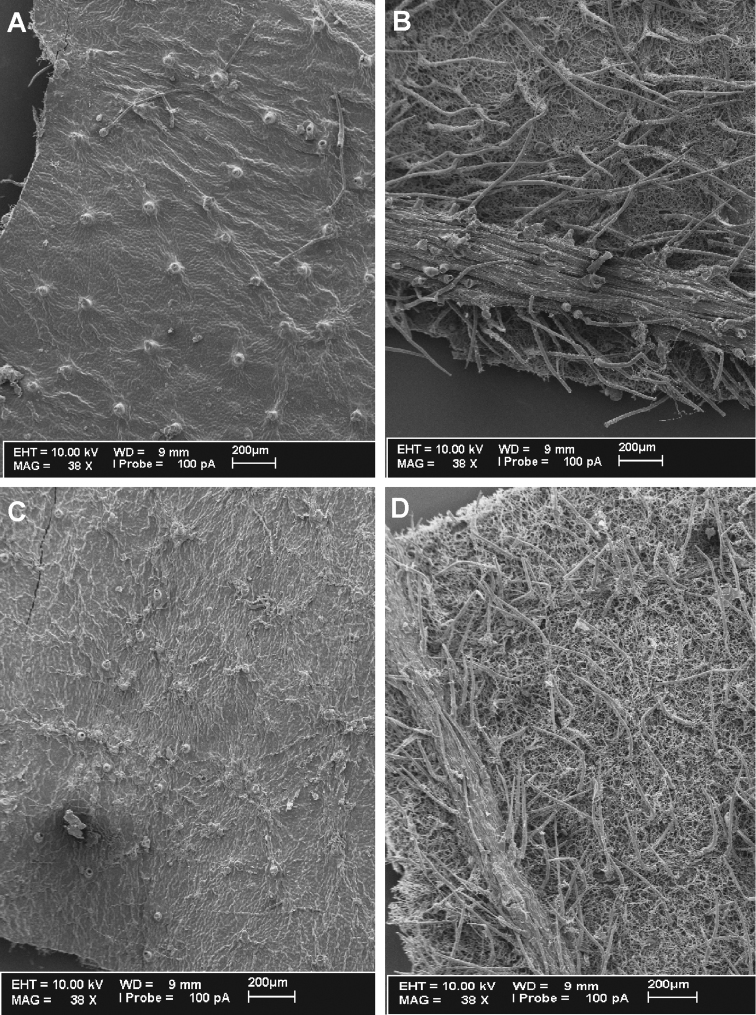
*Cuatrecasanthus* leaf surfaces: **A–B** *Cuatrecasanthus lanceolatus*. **A** Adaxial surface **B** Abaxial surface **C–D** *Cuatrecasanthus sandemanii* **C** Adaxial surface **D** Abaxial surface.

In addition to the diagnostic generic characteristics are features of special interest such as the marginal teeth of the leaves that are incurved and appressed against the abaxial surface in all but one species (*Cuatrecasanthus lanceolatus*; Fig. 1A–B) and the finely branching myceliiform hairs on the abaxial surface of the leaves in all the species (Fig. 1C). In addition, there is variation on the leaf surfaces. The surfaces of the leaves have veins that can be exsculpate (above the surface), insculpate (below the surface), or even with the adaxial leaf surface (Figs 2–4). All but one of the species have veins on the adaxial surface that are even with the surface or slightly insculpate; one species has veins that are deeply insculpate (*Cuatrecasanthus giannasii*) and all six species have veins that are exculpate on the abaxial surface. The style branches are reported on one herbarium label as pale pink almost white; there are no additional data on the color of the styles.

The genus occurs in Ecuador and Peru. The six known species can be distinguished using the following key:

**Table:** 

1	Leaf margins with numerous obvious antrorse teeth not strongly incurved against abaxial surface (may vary in prominence); leaf tips narrowly acute, not abruptly short-acuminate	5. *Cuatrecasanthus lanceolatus*
–	Leaf margins entire or with obscure inturned teeth; leaf tips usually abruptly short-acuminate	2
2	Inflorescence with loose clusters of heads, distinctly exceeding the upper leaves	3
–	Inflorescence with dense clusters of heads, not or scarcely exceeding the upper leaves, with interspersed foliiform bracts	4
3	Leaf blade broadly elliptical or ovate-elliptical; adaxial surface hispidulous with midvein prominently exculpate and otherwise plane	4. *Cuatrecasanthus kingii*
–	Leaf blade lanceolate-elliptical; adaxial surface sparsely covered with appressed minute tricihomes with at least the midvein insculpate	6. *Cuatrecasanthus sandemanii*
4	Adaxial surface of leaf with all veins distinctly insculpate; adaxial surface with few short trichomes, veins and trichomes all whitish; distal leaf margins with incurved teeth pressed against abaxial leaf surface; tips of pappus bristles distinctly broadened	2. *Cuatrecasanthus giannasii*
–	Adaxial surface of leaf with major veins not obviously insculpate, secondary and tertiary veins insculpate; adaxial surface with many prominent stiff trichomes, midvein and trichomes dark brown or yellow; leaf margins with few inturned teeth; tips of pappus bristles not or scarcely broadened	5
5	Abaxial surface of midvein of leaf with dense antrorse pubescence mostly on sides; abaxial surface of lamina covered with mostly appressed, stiff, usually brownish trichomes	1. *Cuatrecasanthus flexipappus*
–	Abaxial surface of midvein of leaf densely hirsute with spreading hairs; abaxial surface of lamina with erect yellowish trichomes	3. *Cuatrecasanthus jelskii*

### 1. Cuatrecasanthus flexipappus

(Gleason) H. Rob., Revista Colomb. Ci. Exact. 17 (65): 210. 1989.

http://species-id.net/wiki/Cuatrecasanthus_flexipappus

Figs 5A 10

Type: Based on *Vernonia flexipappa* Gleason

*Vernonia flexipappa* Gleason, Bull. Torrey Bot. Club 52(5): 186.1925.

#### Type:

Ecuador. Loja: sin. loc., *E. André 2250* (holotype: NY, image US!; isotype: K).

#### Description.

Shrubs or small trees, 1.0–3.0 m tall; *stems* densely pilose with dark brown trichomes, becoming glabrous with age. *Leaves* with petioles 0.5–1.2 cm long; *blades* narrowly to broadly elliptical, mostly 3–9 cm long, 1–3 cm wide, narrowly acuminate at base and apex, margin narrowly but strongly recurved, without evident teeth or with in-turned teeth, adaxial surface dark green, glabrous or with minute appressed pubescence, secondary and tertiary veins insculpate, abaxial surface pale greenish covered with mostly appressed, stiff, brownish trichomes (rarely straw colored) intermixed with less evident whitish prostrate myceliiform branching trichomes, midvein with dense antrorse pubescence mostly on sides; *secondary veins* ca. 5 pairs, spreading from midvein at ca. 45° angles, strongly curved. *Inflorescence* scarcely exceeding vegetative leaves; *branches* densely pilosulous or hirtellous. *Heads* sessile in clusters of 3–7, ca. 10–11 mm tall × 2 mm wide; *involucres* cylindrical to fusiform; bracts mostly deciduous, ca. 15 in ca. 5 series, 1.0–5.5 mm long, ca. 1.2 mm wide, apices short-acute, ovate to narrowly elliptical, yellowish or with reddish median stripe, puberulous to nearly glabrous outside. *Florets* with corollas white to bluish white or lavender, ca. 5.5 mm long, with glandular dots on basal tube and tips of lobes, few small trichomes on lobe tips, tubes ca. 2 mm long, lobes ca. 4 mm long, with some non-glandular trichomes; *anther thecae* ca. 2 mm long. *Achenes* 2.0–2.5 mm long; *pappus* white, of ca. 50 bristles mostly ca. 6 mm long, not or scarcely broadened toward tips. *Pollen* grains 37–47 µm in diam.

**Figure 5.**
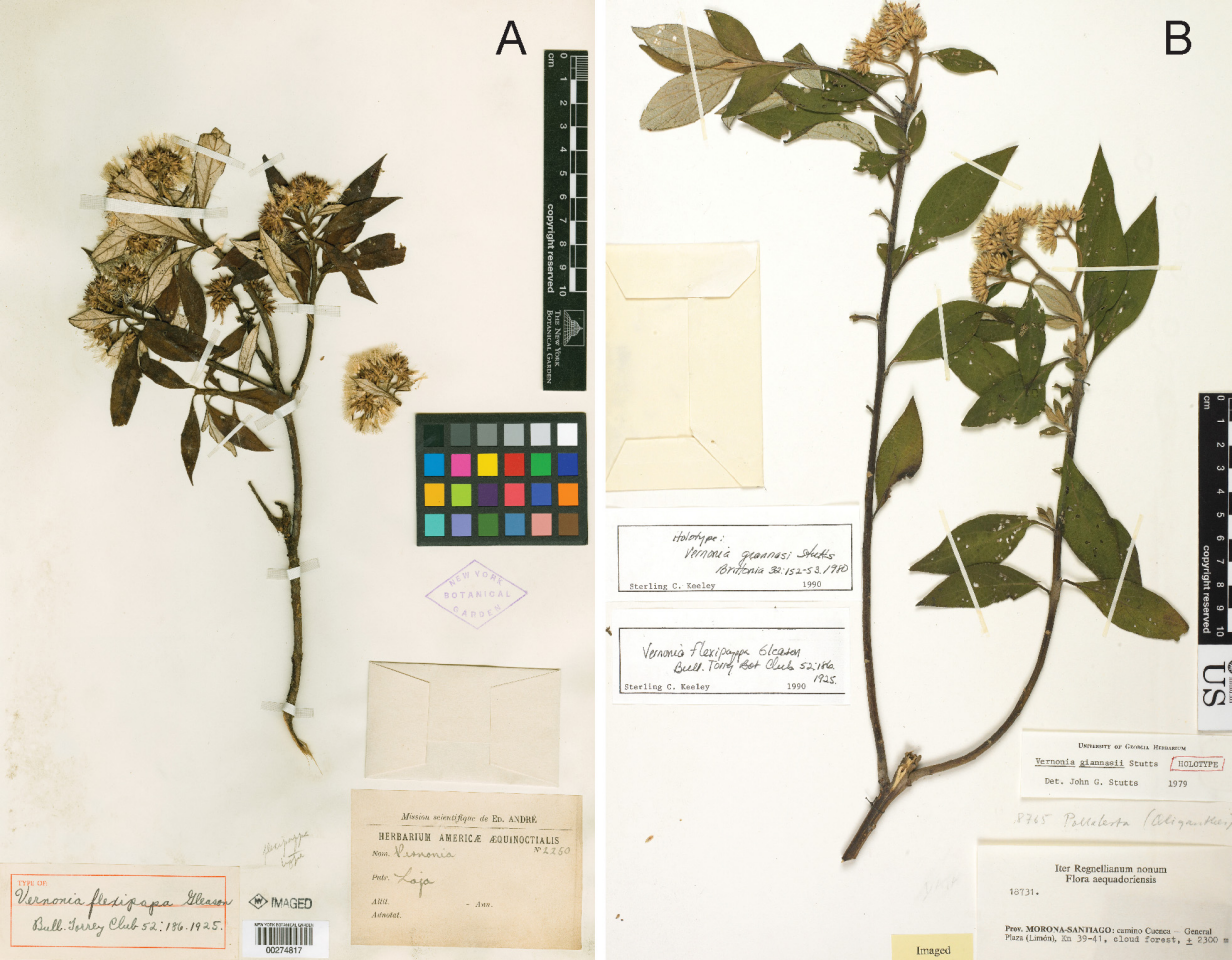
Photographs of *Cuatrecasanthus* types: **A** *Cuatrecasanthus flexipappus*, holotype (NY) **B** *Cuatrecasanthus giannasii*, holotype (S).

#### Additional specimens examined.

Ecuador. **Loja:** along road between Loja and Zamora, ca. km 11 [03°59'0"S, 79°08'16"W, estimated], 2600 m, 2 August 1978, *Zarucchi & Andrade 2304* (US); Carretera Loja–Zamora, km 13, 2500 m [03°59'00"S, 79°07'00"W, estimated], 16 August 1983, *Jaramillo & Winnerskojold 5812* (AAU); Loja–Zamora road, ca. km 15, 03°58'S, 79°08'W, 2400–2700 m, 22–23 April 1984, *Madsen 74081* (AAU, QCA, US); In the páramo of “El Tiro,” located at northern terminus of Podocarpus National Park, 500 m from the Loja–Zamora highway, 03°59'S, 79°08'W, 2940–2970 m, 14 April 1992, *Keating 143* (US); In the páramo and shrub páramo above the Refugio de Cajanuma (Centro de Información), Podocarpus National Park, 04°07'00"S, 79°09'30"W, 2800 m, 31 July 1993, *Keating 409* (US). **Zamora-Chinchipe:** 14.8 km from transit control out of Loja on road to Zamora [03°59'10"S, 79°08'02"W, estimated], 2500 m, 8 July 1983, *Keeley & Keeley 4104, 4105, 4106, 4107, 4108, 4109, 4110, 4111, 4115* (K, US); *Keeley & Keeley 4112, 4114* (US);Zamora, carretera Loja–Zamora, Estación Científica San Francisco, sendero hacia las antenas. Colecciones cerca del Francisco 4, en Transecto 2, 03°58'S, 79°04'W, 3000 m, 29 April 2000; *Freire Fierro 3121* (MO, US).

Peru. **Cajamarca:** Prov. Jaen; E slope of Paso de Huascarai, head of Quebrada Granadillas, 15 km SE of Huancabamba, 05°22'S, 79°20'W, 3000 m, 10 June 1947, *Fosberg 27852* (US). **Piura:** Prov. Huancabamba; Los Llanos to Chorro Blanco (Sapalache – Chiguelas), 2650 m, 5°08.2'S, 79°24.6'W, 19 Oct. 2001, *Sagasteguí, Dillon, Leiva, & Zapata 16781* (F, HUT).

#### Habitat.

Roadside, burned over cloud forest on steep south-facing slope; shrub páramo at 2400–3000 m in elevation.

The species is the most commonly collected member of the genus but apparently is sympatric with both *Cuatrecasanthus kingii* and *Cuatrecasanthus lanceolatus* in the area near the border between Loja and Zamora/Chinchipe. The species is very closely related to the northern Peruvian *Cuatrecasanthus jelskii* (Hieron.) H. Rob. The latter differs most obviously by the densely hirsute abaxial surface of the midvein of the leaves and erect rather than appressed trichomes of the abaxial blade surface. The adaxial leaf surface of the latter also has less strongly insculpate veins.

#### Preliminary conservation status.

Data Deficient

### 2. Cuatrecasanthus giannasii

(Stutts) H. Rob. & V.A. Funk comb. nov.

urn:lsid:ipni.org:names:77121072-1

http://species-id.net/wiki/Cuatrecasanthus_giannasii

Figs 5B 10

Type: Based on *Vernonia giannasii* Stutts

*Vernonia giannasii* Stutts, Brittonia 32(2): 162 (1980).

#### Type.

Ecuador. Morona-Santiago [formerly Santiago-Zamora]: Camino Cuenca, General Plaza (Limon), 39–41, [02°59'S, 78°41'W, estimated], 2300 m, 19 September 1967, *B. Sparre 18721* (holotype: S!).

#### Description.

*Vines or scrambling shrubs*; stems flexuous, densely pilose with long, mostly single-celled trichomes. *Leaves* with petioles 0.4–0.7 cm long; *blades* subchartaceous, elliptical, mostly 3.5–8.5 cm long, 1.0–2.5 cm wide, acuminate at base, acute to short-acuminate at apex, margins appearing entire, narrowly recurved, with inflexed teeth distally, adaxial surface, dark green, bullulate, sparsely short-scabridulous, secondary and tertiary veins insculpate, abaxial surface pale green with thin white cover of myceliiform branched trichomes, minutely pilosulous with pale trichomes on veins, without dark trichomes, all veins and veinlets exsculpate; *secondary veins* ca. 5 or 6 on each side of midvein, spreading at base at 45–50° angles, curved and more strongly ascending near margins. *Inflorescence* terminal and from axils of uppermost leaves, not or scarcely exceeding the leaves, rounded corymbiform; branches short, puberulous. *Heads* sessile with up to 9 clustered in dense glomerules, 8–10 mm tall, ca. 2 mm wide; *involucre* cylindrical or narrowed distally and fusiform, bracts ca. 16 in ca. 5 series, short-ovate to oblong elliptical, 2.0–5.5 mm long, 1.0–1.5 mm wide, apices short-acute, slightly darkened distally, sometimes with reddish median line, glabrous outside. *Florets* with corollas pale lavender, ca. 6.5 mm long, with glandular dots on basal tube and tips of lobes, tubes ca. 2.5 mm long, lobes ca. 4 mm long; *anther thecae* ca. 2.5 mm long. *Achenes* ca. 2.5 mm long; *pappus* white, of ca. 40 capillary bristles ca. 6.5 mm long, distinctly broadened toward tips. *Pollen* grains ca. 40 µm in diam.

#### Additional specimens examined. 

Ecuador. **Loja:** Loja to Zamora, 1876, *André K1152* (F, NY). **Morona-Santiago** [formerly Santiago-Zamora]: Eastern slope of the cordillera, Valley of the ríos Negro and Chupianza (on trail from Sevilla de Oro to Mendez, Tambo Consuelo to Tambo Cerro Negro, [01°49'S, 78°23'W, estimated], 2400-3000 m, 17 December 1944, *Camp E-1619* (NY, US).

The species is known only from Morona-Santiago and Loja, Ecuador, between 2300 and 3200 m in elevation (Fig. 10).

Camp describes the habit as a vine and this character would easily distinguish the species, but the type specimen has no information on the habit and it appears to be a sturdier plant. Only new collections that document the habit will resolve this issue.

#### Preliminary conservation status.

Data Deficient

### 3. Cuatrecasanthus jelski

(Hieron.) H. Rob., Revista Colomb. Ci. Exact. 17 (65): 210 (1989).

http://species-id.net/wiki/Cuatrecasanthus_jelski

Figs 6A 10

Type: Based on *Eremanthus jelskii* Hieron.

*Eremanthus jelskii* Hieron., Bot. Jahrb. Syst. 36(5): 462 (1905), non *Vernonia jelskii* Hieron., Bot. Jahrb. Syst. 36(5): 459 (1905).

Type: Peru. Cajamarca: Prope Shanyn (Quebrada Lejia) [probably not far from Tambillo] [05°40'50"S, 79°16'7"W, Cerro Tambillo, estimated], *Jelski 776* (holotype: B, destroyed, photos F, US! [F neg. 14657]; lectotype, designated here: US!).

*Vernonia shanynensis* MacLeish, Syst. Bot. 9 (2): 135 (1984), nom. nov. for *Eremanthus jelskii*.

Type: Based on *Eremanthus jelskii* Hieron.

#### Description.

*Shrubs or small trees*, 1.0–3.0 m tall; *stems* densely velutinous (short velvety) with dark brown trichomes. *Leaves* with petioles 0.3–0.5 cm long; *blades* narrowly to broadly elliptical, mostly 3–10 cm long, 1–2.5 cm wide, narrowly acuminate at base and apex, margin narrowly but strongly recurved, with few inturned teeth distally, adaxial surface dark green, glabrous or with appressed puberulence, secondary and tertiary veins insculpate, abaxial surface pale green covered with erect, stiff, yellowish trichomes intermixed with less evident whitish prostrate myceliiform branching trichomes, midvein with dense spreading pubescence; *secondary veins* ca. 5–6 pairs, spreading from midvein at 45–55° angles, strongly arched. *Inflorescence* scarcely exceeding vegetative leaves, with intermixed foliiform bracteoles; *branches* densely pilosulous or hirtellous. *Heads* sessile in clusters of 3–7 within larger glomerules, 10−11 mm tall × 1.5−2.0 mm wide; *involucres* cylindrical to fusiform; *bracts* ca. 9–12 in ca. 4 series, 1–5 mm long, ca. 1.2 mm wide, apices short-acute, ovate to narrowly elliptical, yellowish darkened tip, outer bracts puberulous, inner bracts glabrous outside. *Florets* with corollas violet, ca. 6 mm long, with glandular dots on tube and tips of lobes, tubes ca. 2.5 mm long, lobes ca. 3.5 mm long; *anther thecae* deep purple, ca. 3 mm long. *Achenes* 2.0–2.5 mm long; *pappus* white, of 32–ca. 50 bristles mostly ca. 6 mm long, not or scarcely broadened toward tips. *Pollen* grains 37–47 µm in diam.

**Figure 6.**
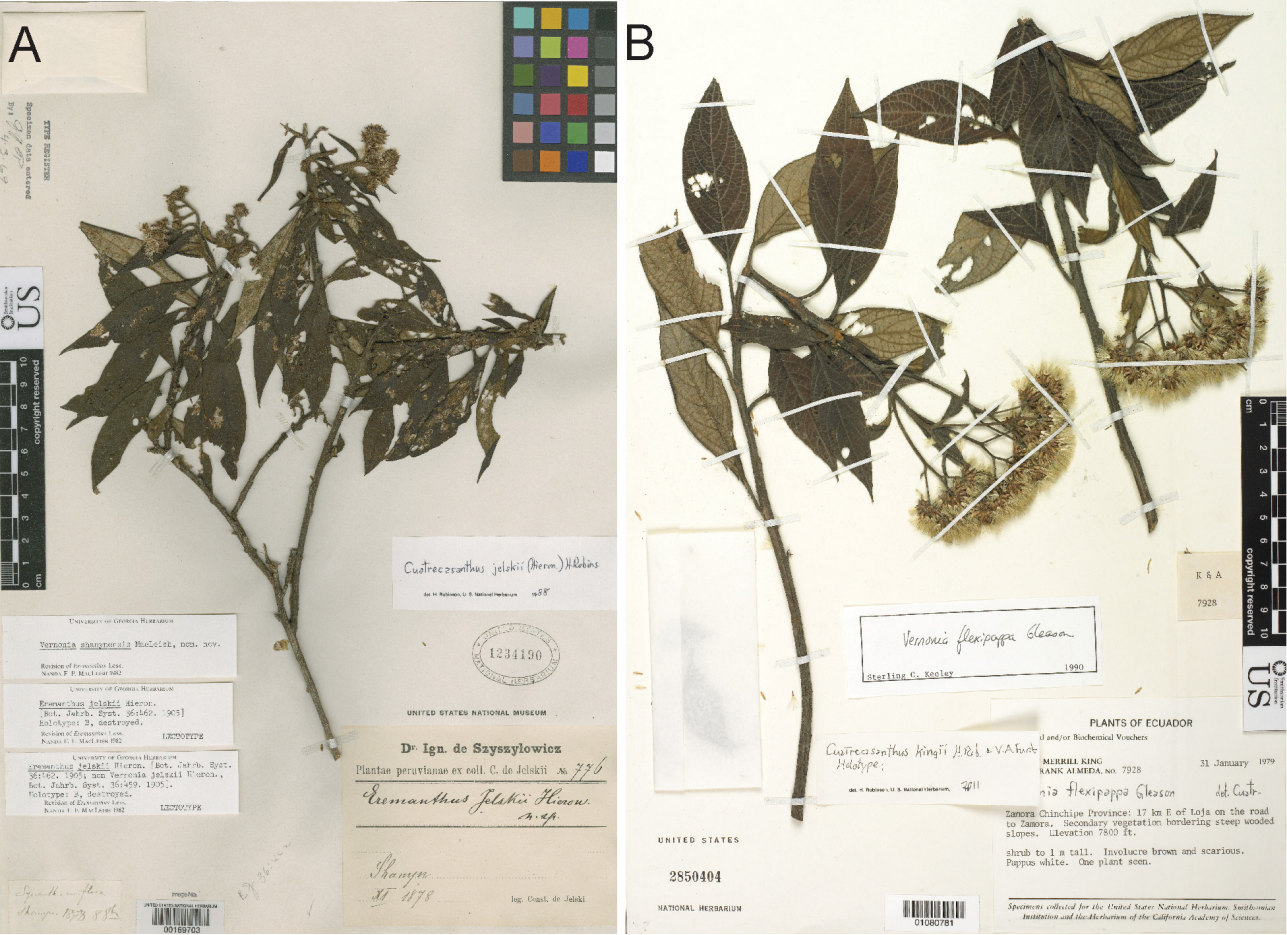
Photographs of *Cuatrecasanthus* types: **A** *Cuatrecasanthus jelskii*, lectotype (US) **B** *Cuatrecasanthus kingii*, holotype (US).

#### Additional specimen examined. 

Peru. **Cajamarca:** Prov. Cutervo; La Pucarilla, entre Sócota y San Andrés [6°16'S, 78°42'W, estimated based on elevation], 2500–2650 m, 24 June 1988, *Sánchez Vega 4868* (CPUN, F, US); 2450 m, 14 November 1986, *Mostacero, Leiva, Mejía, Peláez, & Guevara 1631* (F, HUT); 2 Nov 1991, *Sánchez Vega, Sagastegui & Guevara 5990* (CPUN); Prov. San Ignacio; Cordillera del Condor, Munic. Dist. Huarango, Nuevo Mundo, Caserio Rey del Oriente, arriba, Caseríos Gosén [5°19'S, 78°43'W, estimated for CG but elevation too low] y Pisaguas, 1800 m, 26 July 1997, *Rodríguez & Campos 1816* (MO, US). **Lambayeque:** Prov. Ferrañafe; Paso Upaypecc, Cañaris [6°03'S, 79°16'W, estimated for Cañaris], 3000 m, 25 June 1989, *Llatas Quiroz 2499* (F).

#### Habitat.

*Rodríguez & Campos 1816* was described as having been collected in primary forest. The range in elevation that has been reported is 1800–3000 m (Fig. 10).

This species was the first member of the genus to be described. At the time of its description, a comparison was made to Brazilian species of *Eremanthus*, members of the comparatively distantly related subtribe Lychnophorinae. Herbaria that might hold Jelski collections from Peru and therefore might have additional isolectotypes (according to Chaudhri et al. 1972) are F, KRA, NY and W.

#### Preliminary conservation status.

Data Deficient

### 4. Cuatrecasanthus kingii

H. Rob. & V.A. Funk sp. nov.

urn:lsid:ipni.org:names:77121073-1

http://species-id.net/wiki/Cuatrecasanthus_kingii

Figs 6B 7 10

#### Type.

Ecuador. Zamora–Chinchipe: 17 km E of Loja on the road to Zamora [03°58'53"S, 79°06'31"W, estimated], 7800 ft [2370 m], 31 January 1979, *King & Almeda 7928* (holotype: US!; isotype: CAS).

#### Description.

*Shrubs* to 1 m tall, bases erect or decumbent to rhizomate; *stems* densely lanulate with tawny mostly single-celled trichomes. *Leaves* with petioles 0.8–2.0 cm long; *blades* ovate to elliptical, mostly 3.5–8.5 cm long, 2–3 cm wide; base acuminate, apex short-acuminate, margins appearing entire, narrowly recurved, with incurved teeth distally, adaxial surface dark, epidermal cells often paler in area along veins, surface plane or with slightly insculpate veins, densely hispidulous with stiff trichome bases, abaxial surface densely lanulate to sericeous with tawny trichomes, at surface with dense white cover of myceliumiiform stellate trichomes; *secondary veins* ca. 5 or 6 on each side of midvein, spreading at base at 45–50° angles, curved and more strongly ascending near margins. *Inflorescence* distinctly exceeding reduced distal leaves, with few long ascending branches; *branches* tomentellous with dark hairs. *Heads* sessile and with up to 12 clustered in dense ultimate glomerules, up to 10 mm tall, ca. 2 mm wide; *involucre* cylindrical or narrowed distally and fusiform, bracts brown, ca. 16 in ca. 5 series, short-ovate to oblong elliptical, 2.0–5.5 mm long, 1.0–1.5 mm wide, apices short-acute, slightly darkened distally, sometimes with reddish median line, scarious, glabrous outside. *Florets* with corollas possibly pale lavender, ca. 6.5 mm long, with numerous glandular dots on basal tube and few on tips of lobes, tips of lobes paucipilosulous, tube ca. 2.5 mm long, lobes ca. 4 mm long; *anther thecae* ca. 2.5 mm long. *Achenes* ca. 2.5 mm long; *pappus* white, of ca. 50 capillary bristles ca. 6.5 mm long, not or scarcely broadened toward tips. *Pollen* grains 35–42 µm in diam.

**Figure 7.**
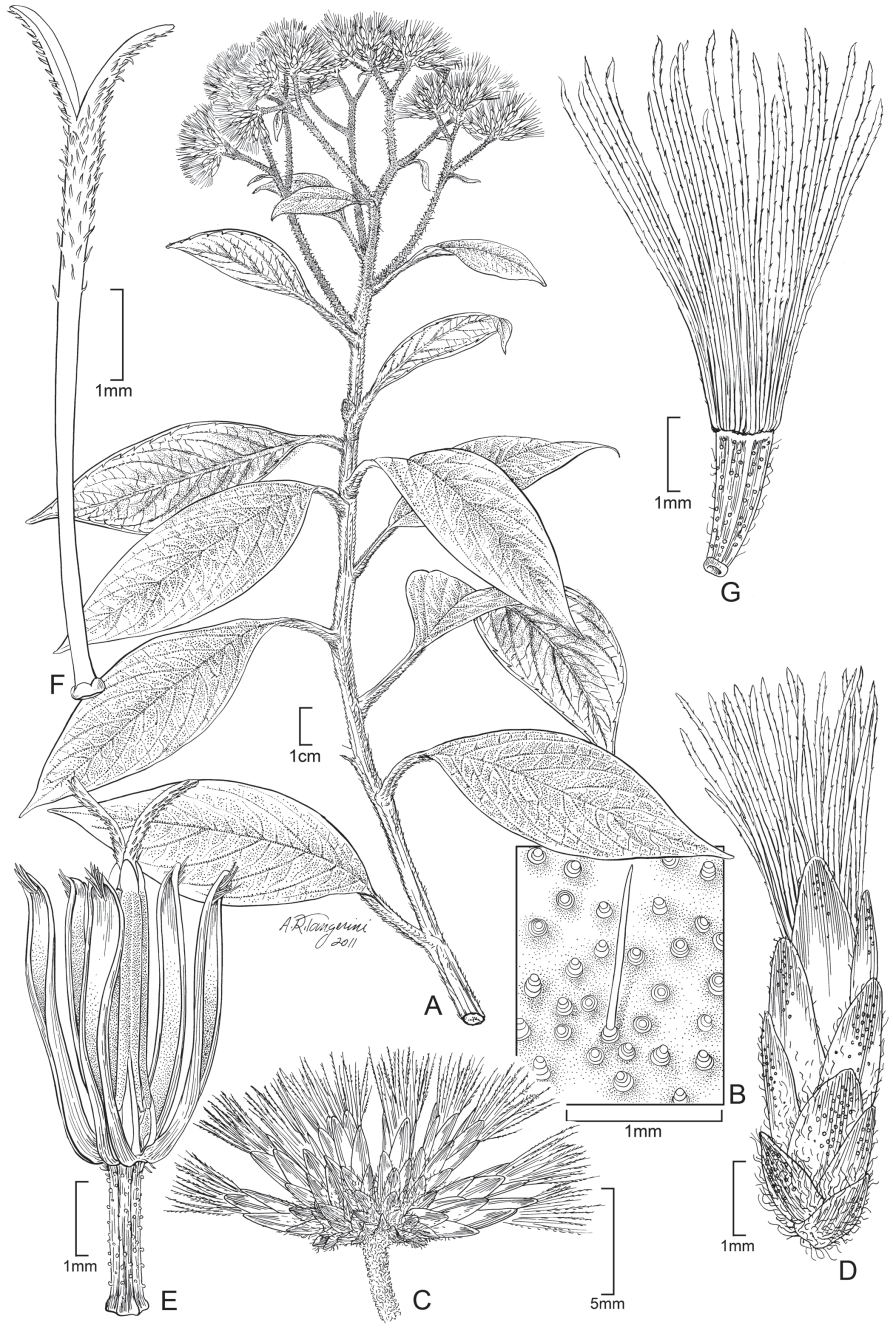
*Cuatrecasanthus kingii*: **A** Habit **B** Detail of adaxial surface of leaf **C** Cluster of heads **D** Single head containing one floret **E** Floret showing corolla lobes divided to base of limb, with thickened margins **F** Style **G** Achene with 8–10 ribs.

#### Additional specimen examined.

Ecuador. **Loja:** 10 km E of Loja on road to Zamora [03°59'07"S, 79°08'16"W, estimated], 2500 m, 31 January 1979, *King & Almeda 7920* (CAS, US).

#### Habitat.

Secondary vegetation bordering steep wooded slopes; wet windswept forested ridge interspersed with pastures at elevations of 2370–2500 m (Fig. 10).

The species has the most broadly elliptical leaf blades of any member of the genus. The most distinctive feature, however, is the mostly flat and hispidulous adaxial surface of the leaves. The distribution is restricted to the area near the pass between the Ecuadorian provinces of Loja and Zamora-Chinchipe (Fig. 10).

#### Preliminary conservation status.

Data Deficient

### 5. Cuatrecasanthus lanceolatus

H. Rob. & V.A. Funk sp. nov.

urn:lsid:ipni.org:names:77121074-1

http://species-id.net/wiki/Cuatrecasanthus_lanceolatus

Figs 8A 9 10

#### Type.

Ecuador. Zamora-Chinchipe:Loja–Zamora, km 20.6, 03°57'S, 79°05'W, 2650 m, 9 August 1997, *G.P.Lewis 3424* (holotype: US!; isotypes: AAU!, GB, K, LOJA, MO, QCA, QCNE).

#### Description.

*Shrubs to small trees* up to 2 m high; *stems* flexuous above, hexagonal, densely pilose with brownish trichomes. *Leaves* with petioles mostly 0.5–1.5 cm long; *blades* lanceolate, broadest at basal 1/3, 4.0–9.5 cm long, 1–3 cm wide, apex distally narrowly acute, not acuminate, margins not or scarcely recurved distally, with marginal teeth projecting upward or outward (may vary in intensity), not inward, adaxial surface dark green, lamina dotted with gland-like persistent or aborted stumps of small scaber, with weakly insculpate veins, abaxial surface gray-green, tawny-pilose, sometimes contorted, denser on veins, with thin grayish prostrate myceliiform branching trichomes; *secondary veins* in 4–5 pairs, strongly ascending. *Inflorescence* distinctly exceeding the reduced distal leaves, main axis and branches mostly deflected at nodes, rounded corymbiform; *branches* tomentellous. *Heads* sessile in clusters of 2–6 congested in larger dense glomerules, 10–11 mm high × 2 mm wide; *involucres* cylindrical or fusiform, ca. 16 in ca. 5 series, 1.0–4.5 mm long, ca. 1.2 mm wide, short-acute, greenish brown, darkened at tips or along midvein distally, glabrous. *Florets* with corollas pink-lilac, ca. 6 mm long, with numerous glandular dots on basal tubes, with a few short hairs at apices of tubes, tubes ca. 2 mm long, lobes ca. 4 mm long; *anther thecae* dark reddish brown, ca. 2.5 mm long. *Achenes* ca. 2 mm long; *pappus* white, of ca. 45 capillary bristles ca. 6.5 mm long, not or scarcely broadened at tips. *Pollen* grains ca. 35 µm in diam.

**Figure 8.**
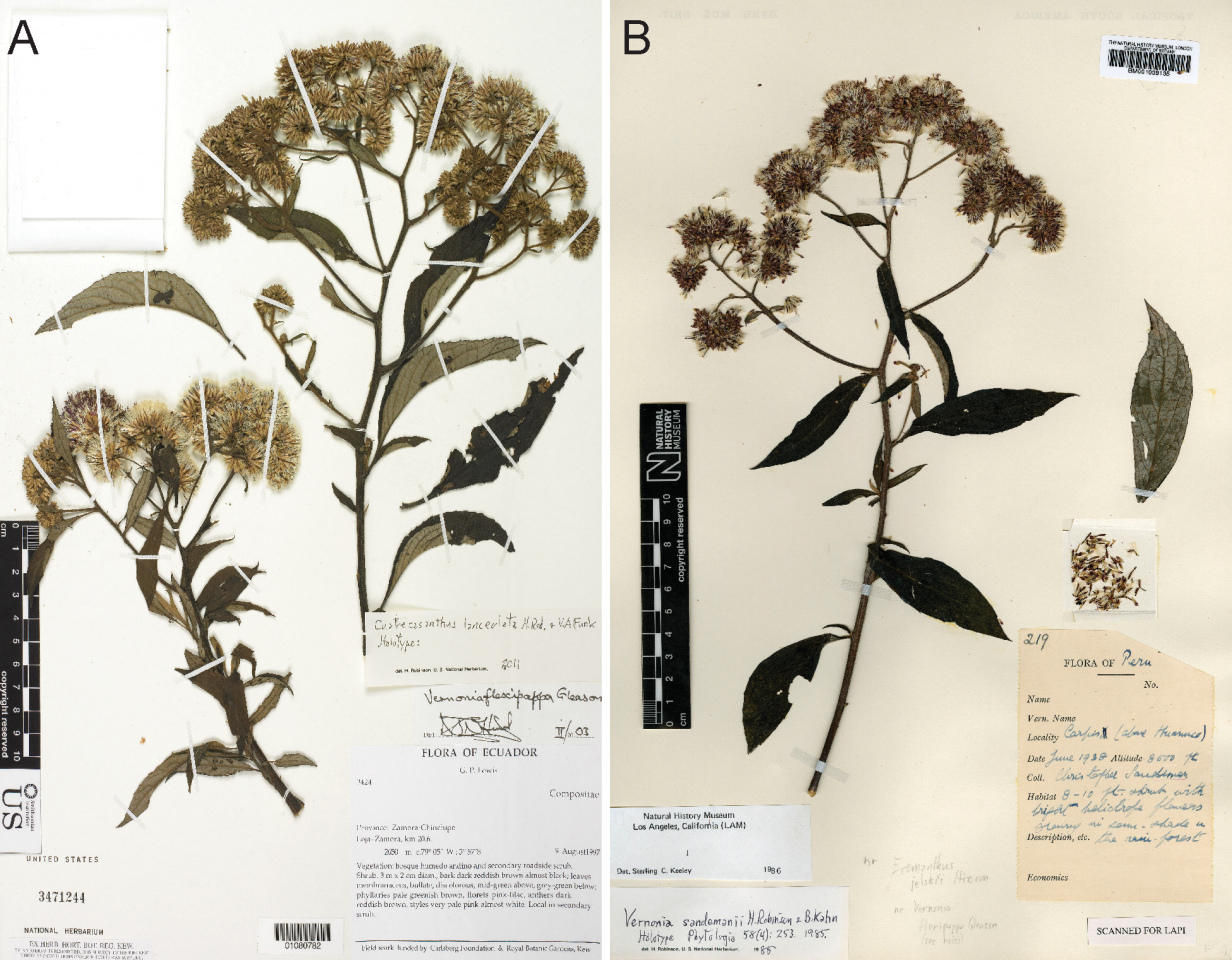
Photographs of *Cuatrecasanthus* types: **A** *Cuatrecasanthus lanceolatus*, holotype (US) **B** *Cuatrecasanthus sandemanii*, holotype (BM).

**Figure 9.**
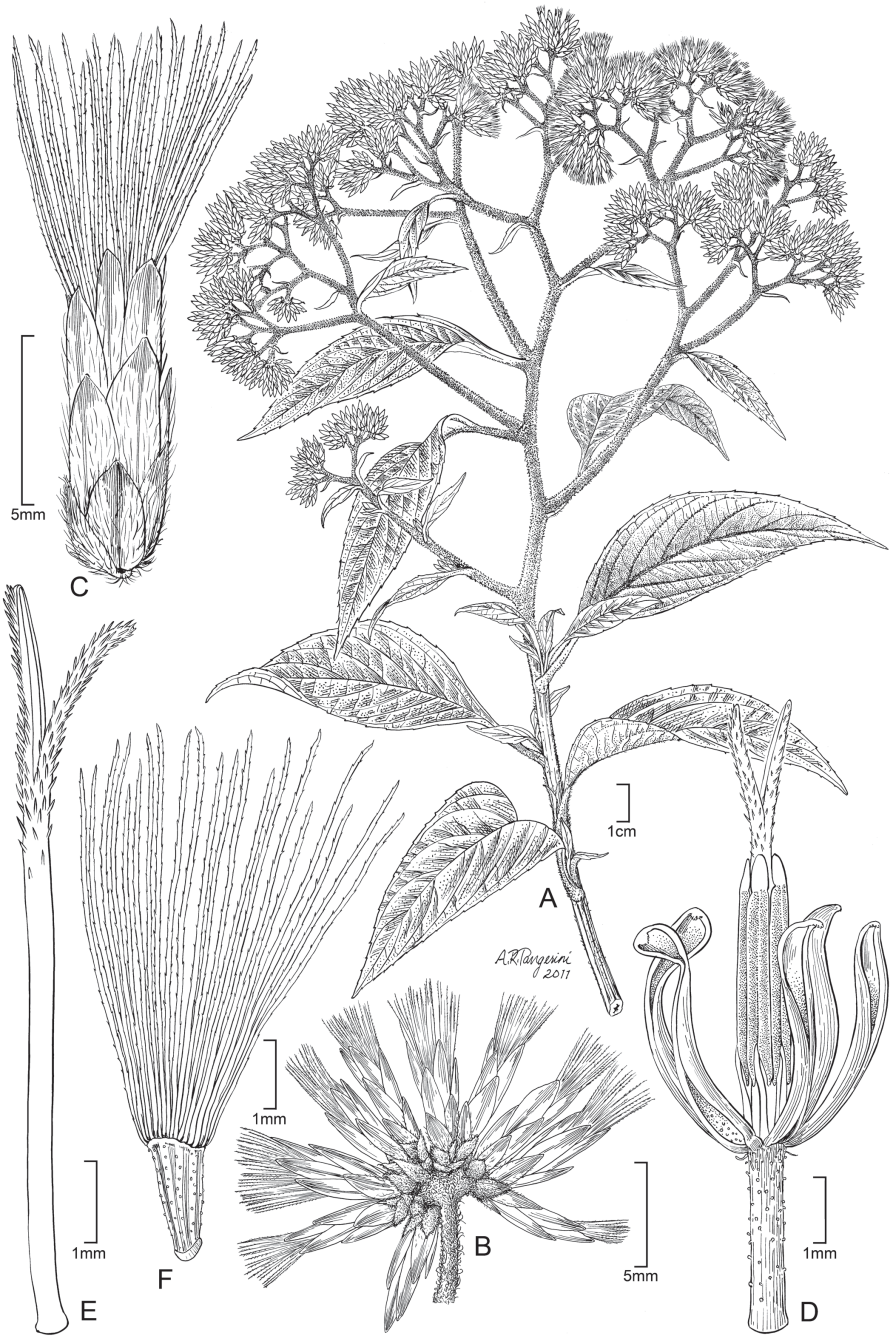
*Cuatecasanthus lanceolatus*: **A** Habit **B** Cluster of heads **C** Single head containing one floret **D** Floret showing corolla lobes divided to base of limb, with thickened margins and apical pubescence **E** Style **F** Achene with 8–10 ribs.

#### Additional specimens examined.

Ecuador. **Loja:** Road to Zamora from Loja, km 12–14, near top of pass, [03°59'6"S, 79°08'23"W, estimated], 2800 m, 28 September 1961, *Dodson & Thien 781* (US-2!).

#### Habitat. 

**Figure 10.**
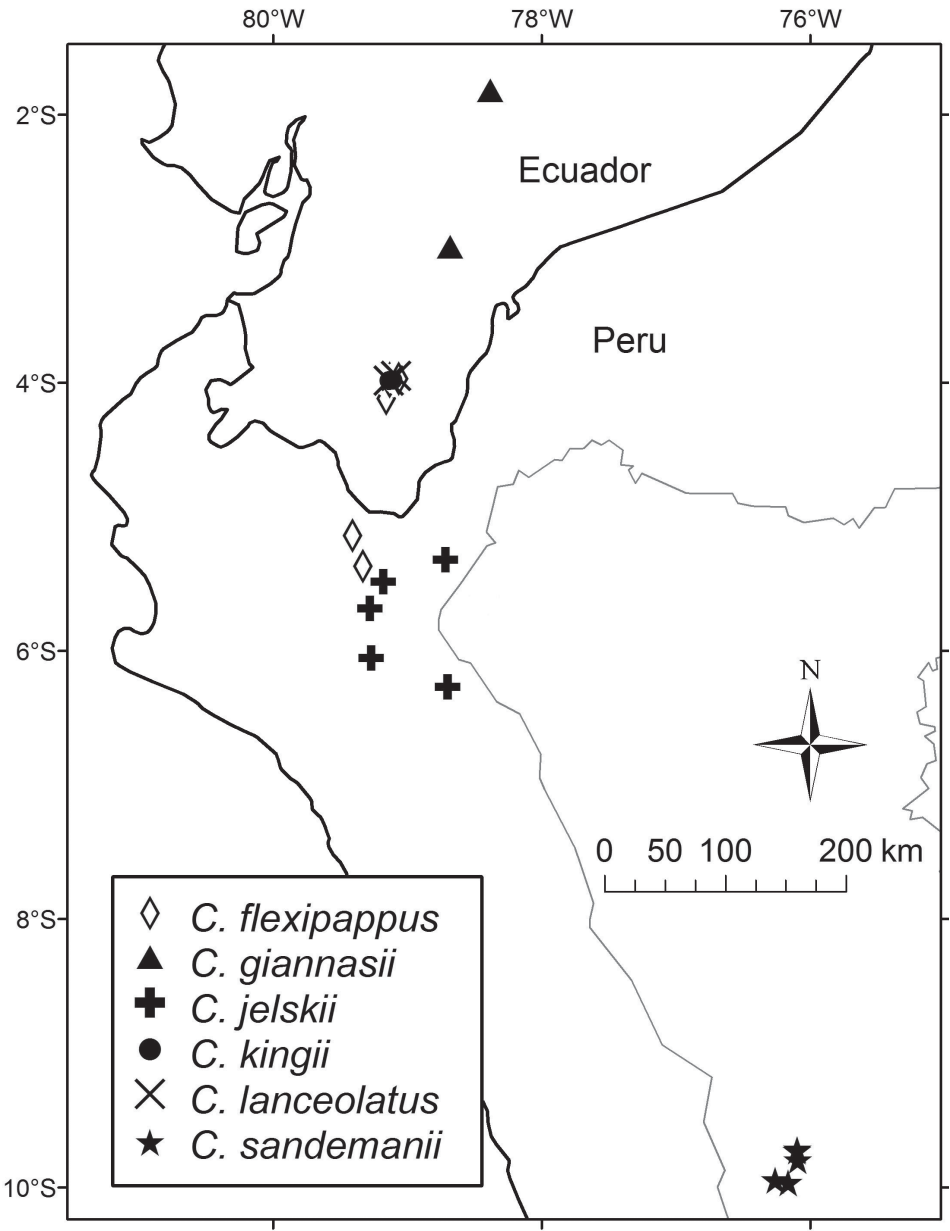
Distribution map of *Cuatrecasanthus* species.

Local in secondary scrub at 2650–2800 m in elevation (Fig. 10).

#### Preliminary conservation status.

Data Deficient

### 6. Cuatrecasanthus sandemanii

(H. Rob. & B. Kahn) H. Rob., Revista Colomb. Ci. Exact. 17 (65): 210 (1989).

http://species-id.net/wiki/Cuatrecasanthus_sandemanii

Figs 8B 10

Type: Based on *Vernonia sandemanii* H. Rob. & B. Kahn

*Vernonia sandemanii* H. Rob. & B. Kahn, Phytologia 58(4): 253 (1985).

#### Type:

Peru. Huánuco: Carpish (above Huánuco) [09°56'47"S, 76°15'51"W, estimated], 8500 ft [2600 m], June 1938, *Cuatrecasanthus Sandeman 219* (holotype: BM!; isotype: K).

#### Description.

*Shrubs to small trees*, to 3.3 m high; *stems* brownish, flexuous above, terete, irregularly appressed pilosulous with short pale trichomes. *Leaves* with petioles mostly 1–3 mm long; *blades* thinly papyraceous (the only species), elliptical, broadest near middle, mostly 7–9 cm long, 1.5–2.7 cm wide, base cuneate, apex narrowly acute to short acuminate, margins narrowly recurved, less recurved at apex, margins becoming shortly serrate distally with few inrolled teeth, adaxial surface dark green, rather shiny, sparsely pilosulous, more densely pilosulous on major veins, primary, secondary and tertiary veins insculpate, abaxial surface whitish, pale sericeous on veins, between major veins whitish tomentellous with prostrate myceliiform branching trichomes; *secondary veins* ascending with5–9 pairs. *Inflorescence* distinctly exceeding the reduced distal leaves, main axis and branches somewhat deflected at nodes, larger foliiform bracts restricted to primary nodes; *branches* densely yellowish sericeous. *Heads* sessile in clusters of 2–6 and clusters congested in numerous larger dense glomerules, 10–12 mm high × 1.5–2.0 mm wide; *involucres* cylindrical or fusiform; involucral bracts greenish brown with exposed parts purplish, ca. 15 in 4–5 series, 1.5–5.0 mm long, ca. 1 mm wide, outer bracts ovate, glabrous to subtomentellous outside, apices rounded to short-obtuse, becoming frayed, linear to narrowly elliptical, mostly glabrous, distally slightly appressed puberulous, short-acute, darkened at tips. *Florets* with corollas violet, ca. 8 mm long, with numerous glandular dots outside, denser on tube and few on tips of lobes, tube 3.5–4.0 mm long, lobes 3.5–4.0 mm long, ca. 0.7 mm wide; *anther thecae* dark reddish brown, ca. 1.3 mm long, bases papillose-fringed; apical appendage oblong, apex rounded. *Achenes* ca. 2 mm long, costae shortly setuliferous, between costae glandular punctate; *pappus* white, of ca. 65 capillary bristles ca. 7 mm long, slightly broadened at tips. *Pollen* grains ca. 45 µm in diam. in fluid.

#### Additional specimens examined. 

Peru. **Huánuco:** Prov. Huánuco, alturas de Carpish, entre Huánuco y Tingo María [09°43'11"S, 76°05'56"W, estimated], 2800 m, February 1940, *Ridoutt s.n.* (USM #11579, US); Carpish, entre Huánuco y Tingo María [09°47'56"S, 76°05'47"W, estimated], 2800–2900 m, 22 August 1946, *Ferreyra 1214* (USM, US), 9 August 1947, *Ferreyra 2347* (US); Alrededor del Tunel de Carpish, 09°43'37"S, 76°06'07"W, 2800 m, 2 November 2001, *Salina 230* (US); Prov. Huánuco, Munic. Dist. Amarilis, Sariapampa [9°58'S, 76°10'W, estimated from elevation], 3100 m, 7 May 1946, *Woytkowski 34295* (F, MO).

#### Habitat:

Near the road in cloud forest and rain forest in semi-shade; 2800–3100 m in elevation (Fig. 10).

#### Preliminary conservation status.

Data Deficient

## Supplementary Material

XML Treatment for Cuatrecasanthus

XML Treatment for Cuatrecasanthus flexipappus

XML Treatment for Cuatrecasanthus giannasii

XML Treatment for Cuatrecasanthus jelski

XML Treatment for Cuatrecasanthus kingii

XML Treatment for Cuatrecasanthus lanceolatus

XML Treatment for Cuatrecasanthus sandemanii
